# PReS-FINAL-2317: Clinical presentation, management and outcome of Kawasaki disease (4 years reviews)

**DOI:** 10.1186/1546-0096-11-S2-P307

**Published:** 2013-12-05

**Authors:** HM Benamer, Halima M Benamer, Brika Brwag

**Affiliations:** 1 pediatric rheumatology, Benghazi children hospital-Libya, Benghazi, Libya

## Introduction

Kawasaki disease(KD) is an autoimmune disease and one of the most common vasculitis of childhood, it has many clinical manifestation but most serious effect is on the heart where it can cause severe coronary artery aneurysms in untreated children

## Objectives

to shade light on our experience on clinical Presentation, management and outcome of KD.

## Methods

Sitting: all medical department of Benghazi children hospital.)) Subject: all the patients who diagnosed as KD during study period(from Jan 2009-dec 201 2).

## Design of study

Retrospective descriptive case series study.

data collected by reviwing their admission files.

## Results

There are 28 patient diagnosed as KD during study period.

Male to female ratio 2.1:1

34% of the cases are atypical KD.

. 79% below 5 years of age...

Peak admissions in October, November, December

The frequency of clinical criteria for diagnosis of KD:

Fever in all the patients (mean duration of fever before admission 8 days), oropharyngeal changes 22 (78%), extremity changes 21 (75%) cervical lymphadenitis 20 (71%), skin rash 18(64%). conjunctivitis 16 (57%).

Other associated symptoms and signs: vomiting 13(46%), diarrhea 11(40%), cough 8(29%), arthritis 2 (7%), hepatomegaly were present in 4(14%), splenomegaly 2 (7%), jaundice 2(7%) no case with meningitis.

Echocardiogram done in 26 patients, 17 (69%) normal, 9 (31%) abnormal coronary arteries.

Regarding treatment: 18 patients receive sandglobulin; aspirin received by 18 patients during admission. no patient receive steroids.

7 patients diagnosed in late stage and 3 patients left LAMA.

Antibiotics used for 17 patients.

## Conclusion

we have significant delay in diagnosis and high rate of coronary aneurysm.

## Disclosure of interest

None declared.

